# Tocilizumab reduces the unmanageable inflammatory reaction of a patient with Aicardi-Goutières syndrome type 7 during treatment with ruxolitinib

**DOI:** 10.1186/s12969-023-00899-4

**Published:** 2023-10-12

**Authors:** Wei Wang, Wei Wang, Siming Peng, Sihao Gao, Meiying Quan, Lijuan Gou, Changyan Wang, Zhixing Sun, Zhuo Li, Dongmei Lian, Hongmei Song

**Affiliations:** Department of Pediatrics, Peking Union Medical College, Peking Union Medical College Hospital, Chinese Academy of Medical Sciences, No.1 Shuaifuyuan Wangfujing Dongcheng District, Beijing, 100730 China

**Keywords:** Aicardi-Goutières syndrome, Treatment

## Abstract

**Background:**

Aicardi-Goutières syndrome (AGS) is a rare hereditary early-onset encephalopathy characterized by upregulation of the type I interferon pathway, poorly responsive to conventional immunosuppression.

**Case presentation:**

We describe a 7-year-old Chinese boy who developed symptoms at the age of 6 months. He presented with a chilblain-like rash, leukopenia, neutropenia, elevated liver enzymesgrowth retardation, microcephaly, elevated acute phase reactants, intracranial calcification and leukodystrophy. At the age of 3 years old, whole-exome sequencing confirmed a de novo heterozygous gain-of-function mutation, c.1016 C > A (p.Ala339Asp), in the IFIH1 gene, and he was diagnosed with AGS7. He was treated with ruxolitinib accompanied by steroids and thalidomide for about four years. The rash, hematological manifestations, and the liver function were all improved, but the erythrocyte sedimentation rate remained consistently elevated until the addition of tocilizumab, a monoclonal antibody against interleukin 6.

**Conclusions:**

Ruxolitinib was not successful in suppressing the inflammatory process, and tocilizumab produced highly encouraging results in reducing the inflammatory reaction of AGS. The study makes a significant contribution to the literature because we may found a potential alternative therapeutic option for AGS.

## Introduction

Aicardi-Goutières syndrome (AGS) is a genetically heterogeneous disorder, originally defined as a progressive encephalopathy with an early age of onset, that is characterized by intracranial calcification, leukoencephalopathy, cerebral atrophy, and inappropriate induction of a type I interferon (IFN)-mediated immune response and belongs to the group of type I interferonopathies, a new heterogeneous group of autoinflammatory disorders [1]. Over time, other consistent features were recognized, such as chilblain-like skin lesions, interstitial lung disease, hypothyroidism, joint contraction, and lupus-like disease. AGS encompasses 9 genotypes (AGS1-9) that involve the genes *TREX1*, *RNASEH2B*, *RNASEH2C*, *RNASEH2A*, *SAMHD1*, *ADAR1*, *IFIH1*, *LSM11*, and *RNU7-1* [2]. These genes encode proteins involved in either aberrant nucleic acid processing or sensing, and mutations in these genes result in chronically enhanced activation of type I IFN signaling.

AGS has been reported to be poorly responsive to conventional immunosuppression in a few studies. According to the retroelement hypothesis of disease pathogenesis, a combination of three nucleoside reverse transcriptase inhibitors (RTIs) (abacavir, zidovudine, and lamivudine) was used to treat patients with mutations in *TREX1*, *RNASEH2A*, *RNASEH2B*, or *SAMHD1* in an open-label pilot study but had no obvious clinical efficacy [3]. Janus kinase inhibitors (JAKis), an example of a strategy to block IFN signaling downstream of a nucleic acid stimulus, have been used in several distinct type I interferonopathies [4]. Definite improvement in AGS-related rash and significant developmental gains have been observed, according to some case reports and open-label pilot studies. However, we have had different experiences with the use of JAKis and have found a potential alternative therapeutic option. Here, we describe one child with IFIH1-related AGS in whom we observed unmanageable inflammation despite the use of ruxolitinib.

## Case report

**Fig. 1:**
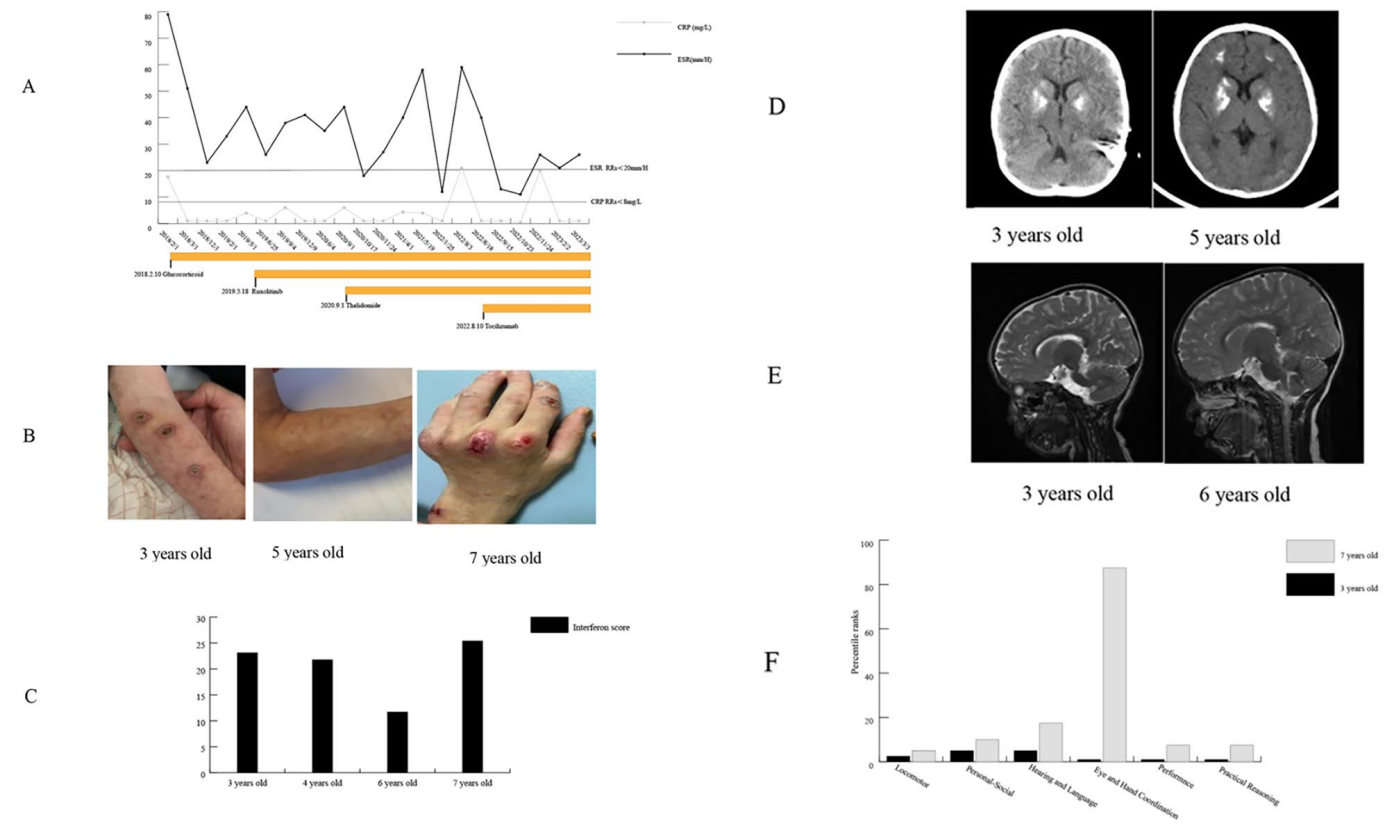
The changes of rash, inflammatory indicators, IS, imaging findings, and GDS during the treatment process. **A** The treatment process including glucocorticoid, ruxolitinib, thalidomide and tocilizumab. C-reactive protein and erythrocyte sedimentation rate values fluctuated with the different medications. **B** Rashes of varying severity at different ages. The first picture (3 years old) was the sinus that formed after one year of treatment with glucocorticoids. The second picture (5 years old) was the skin manifestation after two years of treatment with ruxolitinib, and the last picture (7 years old) was the rash when the disease relapsed. **C** The interferon score values during the treatment. **D** Cranial computed tomography images. **E** Cranial magnetic resonance imaging. **F** Griffiths Developmental Scale during the treatment

A male infant was born to unrelated Chinese parents at full term. His birth weight was 3,000 g, birth length was 50 cm, head circumference was 34.5 cm, and Apgar scores were 10 and 10 at 1 and 5 min, respectively. The development of his disease has been published previously in the Journal of Evidence-based Pediatrics [5], so this paper focuses on the treatment process(Fig. 1A).

At 6 months of age, the patient was hospitalized for a chilblain-like rash distributed on the face, ears, arms and legs that worsened when the weather was cold, accompanied by irregular fever. Blood examinations revealed leukopenia (2.61 *10^9/L)(range 3.5–9.5*10^9/L), neutropenia (0.82*10^9/L)(range 2-7.5*10^9/L), elevated liver enzymes (alanine aminotransferase (ALT) 60 U/L(range 7-40U/L), aspartate aminotransferase (AST) 91 U/L)(range 13-35U/L), and increased inflammatory parameters erythrocyte sedimentation rate (ESR) 79 mm/H (range 0-20 mm/H), C-reactive protein (CRP) 17.6 mg/L(range 0-8 mg/L)), and interleukin 6 (IL-6) 13.6 pg/ml (range < 8 pg/ml). Positive antinuclear antibody(ANA) (1:100) was detected, autoimmune hepatitis was suspected in other hospital althought negtive anti-smooth muscle antibody and anti-LKM antibody. At 1.5 years of age, he was treated with glucocorticoids at a dose of 0.5 mg/kg. However, the rash worsened, sinuses formed on the face and upper limbs(Fig. 1B), and the abnormal blood test indicators did not return to normal levels.

At 3 years old, the patient was hospitalized in our hospital. His admission physical examination showed growth retardation and microcephaly. The neurological examination showed no signs of extrapyramidal involvement. The Griffiths Developmental Scale (GDS) showed global developmental retardation, including locomotor, personal-social, hearing and language, eye and hand coordination, performance, and practical reasoning. Laboratory testing showed subclinical hypothyroidism. Serum immunoglobulins and lymphocyte subsets were normal. A computed tomography (CT) scan of the chest was performed and displayed mild interstitial lung disease. A CT scan of the head showed obvious calcification in the bilateral basal ganglia, and cranial magnetic resonance imaging (MRI) showed patches of slightly longer T2 signals in the bilateral cerebral hemisphere(Fig. 1D and E). White matter volume was normal. Whole-exome sequencing confirmed a de novo heterozygous gain-of-function (GOF) mutation, c.1016 C > A (p.Ala339Asp), in the *IFIH1* gene (NM_022168.4). We have done sanger sequencing for this variant and used SIFT, PolyPhen2, and MutationTaster to predicte the pathogenicity of the variant, and the results were damaging, probably damaging, and disease causing respectively. The CADD score was 27.8. The expression of six interferon-stimulated genes (ISGs), including IFIT1, IFI27, IFI44L, ISG15, SIGLEC1, and RSAD2, was measured by quantitative PCR, and the median fold change, when compared with the median of healthy controls, was used to create an interferon score (IS) for each patient. Scores higher than the mean of controls plus two SD (range > 2.441) were designated as positive [1].IS of our case was 23.11. A diagnosis of AGS7 was made. Therefore, treatment with ruxolitinib, a JAKi, was initiated at a dose of 0.5 mg/kg. The rash improved, and AST, ALT, white blood cell count, and thyroid function indicators returned to normal levels. The IS decreased to 21.78. ESR consistently increased after a transient decline despite increasing the dose of ruxolitinib to 0.7 mg/kg.

At 4.5 years old, thalidomide was added at a dose of 12.5 mg once daily while maintaining treatment with glucocorticoids and ruxolitinib. Three months later, the dose of thalidomide was increased to 25 mg once daily. Only two ESR measurements decreased to normal levels; otherwise, ESR was elevated, ranging from 14 mm/H to 60 mm/H. The IS decreased to 11.707. During this period, infection was ruled out, and we tried to adjust the doses of glucocorticoids and ruxolitinib, but the systemic inflammatory response was difficult to inhibit.

At 6.5 years old, tocilizumab was started at 160 mg intravenous(IV) every 2 weeks, and ESR decreased to normal quickly. However, after the second administration of tocilizumab, ALT increased to 69 U/L, creatine kinase–myocardial band increased to 77.3μg/L(range ≤5.0μg/L) and cardiac troponin I increased to 116.9ng/L(range ≤ 54ng/L), all of which were believed to be caused by the side effects of tocilizumab when other reasons had been excluded. Therefore, the dose of tocilizumab was decreased to 80 mg IV every 2 weeks. The ESR values fluctuated between 18 mm/H and 26 mm/H after this adjustment.

At 7 years old, despite combination therapy with glucocorticoids, ruxolitinib, thalidomide and tocilizumab, the chilblain-like rash recurred and was mainly distributed on the hands. The joints of the fingers gradually flexed and could not be straightened. Additionally, progressive joint contractures in the interphalangeal joints of both hands were observed. Upon rehospitalization for assessment, routine blood tests, biochemical tests, myocardial enzyme tests, and thyroid function tests were all within normal ranges. ESR was measured at 26 mm/H, and the IS increased to 25.11. Lymphocyte subsets revealed a decreased count of CD4 cells at 195 cells/μl (range 686–1358 cells/μl), while immunoglobulin levels remained within the normal range. Joint ultrasonography and MRI did not reveal any joint effusion, synovial thickening, or other structural abnormalities. Furthermore, a comprehensive evaluation of the neurological system was conducted. The GDS indicated a significant improvement in various developmental aspects compared to previous assessments(Fig. 1F). However, there was progressive intracranial calcification, and no signs of amelioration were observed in the leukodystrophy(Fig. 1D and E).

## Discussion

We have detailed the challenging treatment process of a diagnosed case of AGS7 characterized by uncontrolled inflammatory reactions. Despite the administration of various drugs, such as steroids, ruxolitinib, and thalidomide, the ESR remained consistently elevated. Ultimately, after the addition of tocilizumab, the ESR decreased to normal levels. The rash reappeared after a four-year remission, accompanied by new joint symptoms. There was an improvement in cognitive testing, but no significant changes were observed in intracranial imaging.

Ruxolitinib is a commonly used JAK1/2 inhibitor. There is mounting evidence of the possible beneficial effect of JAKis in subjects with AGS. Ruxolitinib has also been shown to be effective in the treatment of lesions in patients with familial chilblain lupus with *TREX1* mutations [6]. Additionally, two AGS2 patients with a severe developmental delay treated with ruxolitinib showed an improvement in psychomotor retardation and a reduction in the IS [7]. In addition, a patient with AGS7 who did not respond to therapy with intravenous immunoglobulin and corticosteroids started ruxolitinib 5 mg/day and showed clinical improvement, recovery of neuromotor skills, increase in neuropsychiatric function scales and improvement of neuroradiological findings [8]. In this study, our patient also showed relief of his rash, which lasted for 4 years. But after a four-year remission, he experienced a new joint symptom. Some reasons may explain this phenomenon. Firstly, AGS could affects the joints. The disease might progressed slowly due to the long-term unmanageable inflammatory reaction. Second, drug resistance might developed after long-term treatment with ruxolitinib, but it cannot be verified at present. Other JAKis, such as baricitinib and tofacitinib, may be alternative drugs. We use GDS to assess the case’s cognitive and perceptual skills, including locomotor (A), personal and social skills (B), hearing and language (C), hand-eye coordination (D), performance (E), and practical reasoning (F). The raw scores of the six subscales were converted to the corresponding percentiles and functional age standard by the Chinese norm and what present in our article is percentiles. Our case showed an improvement in GDS but mild signs of improvement in the neurological picture. Most likely, the clinical picture was dominated by severe central nervous system damage, which is unique to AGS and is almost irreversible.

Elevated inflammatory indicators occur in approximately 53% of AGS patients [9]. The relationship between ISG and ESR is undefined. However, according to the data of this case, in his 3, 4, 6, and 7 years of age, the level of ESR was 25 mm/H, 35 mm/H, 59 mm/H, and 26 mm/H respectively, while the IS testing at the same time was 23.1, 21.7, 11.7, and 25. Although IS can reflect the activation of type I interferon signaling pathway, but it may be affected by viral infection and age. So we pay attention to ESR at the same time. The changing trends were not consistent.The elevated ESR of our patient did not decrease to normal until the use of tocilizumab, which indicated a poor effect of ruxolitinib in inhibiting inflammation. This phenomenon has not been reported in the literature because most of the patients in the reports did not have elevated acute phase reactants. In studies on ruxolitinib treatment for other type I interferonopathies, such as STING-associated vasculopathy of infantile onset (SAVI), the efficacy varied. Malak et al. reported two siblings diagnosed with SAVI treated with ruxolitinib over a span of 6 months, and they exhibited a significant improvement in lung and dermatological symptoms but a decline in ESR (60 mm/H→30 mm/H and 54 mm/H→44 mm/H) [10]. Stefano Volpi detailed three patients with SAVI, and after treatment with ruxolitinib, they observed improvements in respiratory function and diverse changes in ESR (55 mm/H→30 mm/H, 26 mm/H→23 mm/H, and 76 mm/H→79 mm/H) [11]. Tofacitinib, a JAK1/2/3 inhibitor, has shown some efficacy in the treatment of SAVI [12, 13]. Therefore, JAKis may have limited efficiency in suppressing the inflammatory process in type I interferonopathies. Alternative signaling pathways to the type I interferon pathway were considered in AGS patients. Additionally, in vitro cell experiments have demonstrated that after IFN-I stimulation of astrocytes, other signaling pathways are activated, and upon the removal of IFN-I stimulation, other inflammatory factors persist. It is also thought that the activation of other signaling pathways may contribute to neurological pathology [14]. Therefore, there is an urgent need to discover new signaling pathways and target-specific drug treatments.

IL-6 has a broad effect on cells of the immune system and has context-dependent pro-and anti-inflammatory properties and is now regarded as a prominent target for clinical intervention. Tocilizumab, a monoclonal antibody against IL-6 is used in the treatment of juvenile idiopathic arthritis and autoinflammatory diseases, such as familial Mediterranean fever, cryopyrin-associated periodic syndrome, and tumor necrosis factor receptor-associated periodic syndrome [15]. The possible role of IL-6 in the pathogenesis of AGS-related interferonopathies remains to be elucidated. In 2000, Harcourt JL et al. demonstrated that although IFNα does not directly induce IL-6 expression, pretreatment with IFNα synergistically enhances IL-6 expression in response to dsRNA [16]. The type I interferon signaling pathway in AGS patients is continuously activated. Therefore, it is speculated that AGS patient more likely to produce IL-6 when the body sense dsRNA, such as virus, which lead to severe inflammatory reaction and couldn’t be suppressed by JAK inhibitors. Henrickson and Wang started tocilizumab treatment in an AGS5 patient with systemic and neurological signs consistent with multifocal stenosis and aneurysms within large arteries, moyamoya, chronic ischemia, and early-onset strokes. The treatment resulted in regression of cerebral vasculopathy and decreases in inflammatory markers [17]. Our case, who had a elevated IL-6 and persistent unmanageable inflammatory reaction, the second treated case with promising effects. A larger series of patients needs to be explored to prove the efficacy of this therapeutic option.

In the fifth year of treatment, despite treatment with glucocorticoids, ruxolitinib, thalidomide, and tocilizumab, the patient experienced recurrent chilblain-like rash and joint contractures in the hands, along with an elevation in IS without cause. Lambe J et al. reported a relapsing-remitting clinical course in female patients with AGS2 [18]. This situation inevitably limits our knowledge of the natural history of AGS, which makes it difficult to establish clearly whether the clinical and radiological improvements were causally related to pharmacological treatment.

## Conclusion

In summary, this study describes the 5-year follow-up management of a patient with AGS type 7. Ruxolitinib did not exhibit significant efficacy in alleviating the systemic inflammatory process in AGS, while tocilizumab may be a potential therapeutic option to decrease the inflammatory reaction. Additionally, it is important to investigate the natural course of AGS, which is the foundation for judging the effect of clinical intervention.

## Data Availability

All data generated or analysed during this study are included in this published article.

## List of abbreviations

AGS: Aicardi Goutières syndrome
ALT: Alanine aminotransferase
AST: Aspartate aminotransferase
CRP: Reactive protein
CT: Computed tomography
ESR: Erythrocyte sedimentation rate
GDS: Griffiths Developmental Scale
GOF: Gain-of-function
IFN: Interferon
IL-6: Interleukin 6
IS: Interferon score
IV: Intravenous
JAKi: Janus kinase inhibitor
MRI: Magnetic resonance imaging
RTI: Nucleoside reverse transcriptase inhibitor
SAVI: STING-associated vasculopathy of infantile onset
